# Regulation of the BRCA1 gene by an SRC3/53BP1 complex

**DOI:** 10.1186/1471-2091-12-50

**Published:** 2011-09-13

**Authors:** Dale Corkery, Gobi Thillainadesan, Niamh Coughlan, Ryan D Mohan, Majdina Isovic, Marc Tini, Joseph Torchia

**Affiliations:** 1 Department of Oncology, London Regional Cancer Program and the Lawson Health Research Institute, 790 Commissioners Rd, London, Ontario N6A 4L6 Canada; 2 Department of Biochemistry, Schulich School of Medicine & Dentistry, University of Western Ontario, London, Ontario N6G 2V4 Canada; 3 Department of Physiology and Pharmacology, Siebens-Drake Medical Research Institute, Schulich School of Medicine & Dentistry, University of Western Ontario, London, Ontario N6G 2V4 Canada

## Abstract

**Background:**

Steroid Receptor coactivator 3(SRC3) is an oncogene and a member of the SRC family of nuclear receptor coactivator proteins that mediate the transcriptional effects of nuclear hormone receptors as well as other transcription factors.

**Results:**

We have used protein purification and mass spectrometry to identify the 53BP1 tumour suppressor as a novel SRC3-associated protein. Copurification was demonstrated using multiple antibodies, and was not dependent on DNA damage suggesting that SRC3 is not directly involved in the DNA damage response. However using chromatin immunoprecipitation(ChIP) and siRNA knockdown, we have demonstrated that both SRC3 and 53BP1 co-occupy the same region of the BRCA1 promoter and both are required for BRCA1 expression in HeLa cells.

**Conclusions:**

Our results suggest that both 53BP1 and SRC3 have a common function that converge at the BRCA1 promoter and possibly other genes important for DNA repair and genomic stability.

## Background

The steroid receptor coactivator 3 (SRC3) (also known as p/CIP/AIB1/ACTR/NCoA3) is a member of the SRC family of proteins which bind to nuclear hormone receptors, and other transcription factors, to promote coactivator complex assembly at target genes [1-6]. This is accomplished through direct protein interactions mediated by several structural domains conserved among all of the SRC family members. These domains include a basic helix-loop-helix Per/ARNT/SIM (bHLH-PAS) domain, a nuclear receptor interacting domain which consists of three leucine-rich motifs with the consensus amino acid sequence LxxLL (X = any amino acid and L = leucine), and two transcriptional activation domains (AD1 and AD2) within the carboxy terminus of SRC3. The AD1 domain interacts directly with various protein acetyltransferases such as CBP/p300, or p/CAF/GCN5 and is absolutely essential for SRC3-mediated transcriptional activation [1,2,7-10]. A second transactivation domain (AD2) serves as an interaction surface for several members of the protein arginine methyltransferase (PRMT) class of enzymes such as the coactivator associated arginine methyltransferase 1 (CARM1, also known as PRMT4) and PRMT1 [11,12]. CARM1 interacts directly with the AD2 domain of all the SRC proteins and co-transfection assays using transient or stably integrated reporter genes have shown that individual SRC proteins can synergize with p/300/CBP and CARM1 suggesting that the coordinated recruitment of acetyltransferases and methyltransferases by SRC3 may be complimentary to one another and represent essential activating steps in nuclear receptor-dependent gene transcription [13].

SRC3 function is also regulated by multiple cytoplasmic signalling events. Recent studies have identified numerous post-translational modifications within SRC3 such as phosphorylation, acetylation, ubiquitination and methylation [14-18]. Consequently, the type and specific sites of covalent modifications in SRC3 determine the affinity for the liganded-NR, as well the association with different coactivating partners, resulting in the formation of diverse multimeric complexes which are believed to regulate distinct gene expression programs.

SRC3 is located within a region of chromosome 20 that is often amplified in breast and ovarian cancer [3]. Several studies have confirmed that SRC3 is amplified in a significant fraction of breast tumours, with amplification frequencies of approximately 10% [19-21]. A positive correlation has also been found between amplification of SRC3 and increased expression of the HER2 oncogene [22-24]. Patients whose tumours expressed high levels of both p/CIP and HER-2 exhibited the poorest survival rates and the worst responses to tamoxifen therapy. In MCF-7 cells, downregulation of SRC3 using small interfering RNA (siRNA) decreased βestradiol-dependent cell proliferation and inhibited expression of several ERα targets [25,26]. Importantly, decreased expression of SRC3 in MCF-7 cells is associated with a significant reduction in estrogen-dependent colony formation and tumour growth in nude mice [25,27]. Collectively, these results suggest that SRC3 is required for maximum ER activation, and amplification and overexpression of SRC3 is a contributing factor that promotes ER-dependent signalling in the mammary gland and in breast cancer. Transgenic mice overexpressing SRC3 under the control of the MMTV promoter have provided further evidence for its oncogenic capacity [28]. The mammary glands of SRC3 transgenic mice were associated with increases in cell proliferation, reduced apoptosis and a high tumour incidence. Furthermore, increases in IGF-I mRNA levels and activation of IGF/AKT/mTOR signalling pathway were also found in the mammary gland tumours.

In the present study, we purified SRC3 from Hela cell nuclear extracts and using mass spectrometry, we have identified the DNA damage response protein 53BP1 as a novel SRC3-associated protein. The colocalization of SRC3 and 53BP1 also included CBP based on copurification, and was restricted to the nuclear compartment. Colocalization of SRC3 and 53BP1 was not dependent on DNA damage suggesting that SRC3 likely plays no direct role in the DNA damage response. However, using both chromatin immunorecipitation (ChIP) and siRNA knockdown, we have determined that SRC3 and 53BP1 co-occupy the same region of the BRCA1 promoter and are both required for BRCA1 expression in Hela cells. These results suggest that the 53BP1/SRC3 complex may play a role in modulating the DNA damage response by regulating the expression of a subset of target genes important for DNA repair.

## Results

**Figure 1:**
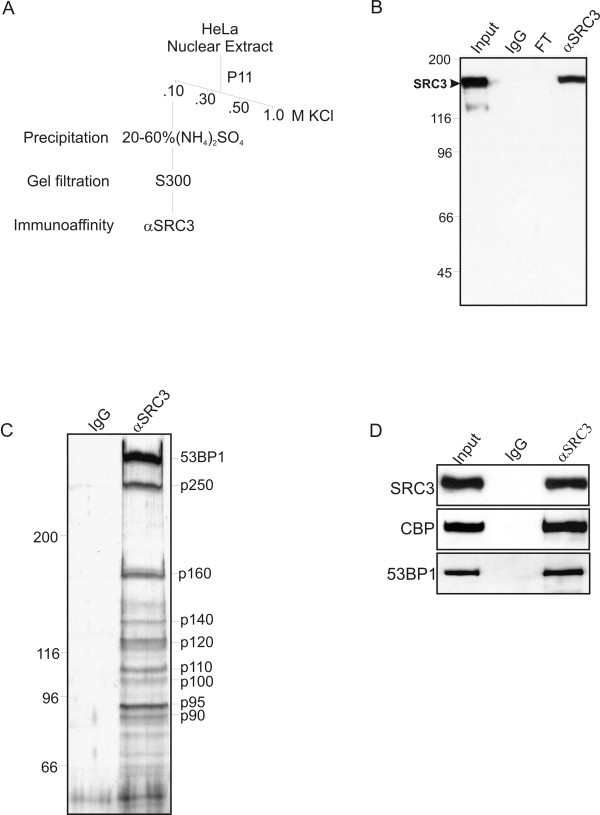
**Purification of SRC3 from HeLa cell nuclear extracts**. (A) Purification scheme for the SRC3 protein (B) Western blot analysis following affinity purification of SRC3. The SRC3-containing gel filtration fractions were pooled and passed through an IgG immunoaffinity column or an αSRC3 immunoaffinity column; FT: flowthrough fraction. A 10 μl aliquot of the purified proteins were separated by SDS-PAGE gel and western blotting was performed using α SRC3 antibody. (C) Silver stained SDS-PAGE gel of purified SRC3 and its associated proteins. IgG; affinity purification using a rabbit IgG affinity column. (D) Western blotting using selected antibodies. 10-20 μl aliquots of either control or affinity-purified SRC3 were separated by SDS-PAGE gel, transferred to nitrocellulose and probed with either SRC3, CBP or 53BP1 as indicated on the left.

To purify SRC3-associated proteins we combined conventional and immunoaffinity chromatography using an αSRC3 antibody [1] (Figure 1A). HeLa cell nuclear extracts were prepared and fractionated on a P11 phosphocellulose column using a buffer containing increasing salt concentrations. Western blot analysis of the eluates derived from the P11 column indicated that SRC3 eluted in buffer containing 0.1 M KCl (data not shown). The SRC3-containing fraction was then subjected to further purification using gel filtration chromatography followed by affinity purification using an α SRC3 antibody crosslinked to Protein A Sepharose (Figure 1B). SDS-PAGE analysis followed by silver staining detected several other proteins ranging in molecular weight from 43 to 350 kDa (Figure 1C). The identity of SRC3-associated proteins was found to vary between different purifications with the exception of the band migrating at approximately 300 kDa which was consistently observed in all of the independent purifications performed and was identified by mass spectrometry as the DNA damage response protein p53 binding protein 1 (53BP1). To confirm our mass spectrometry findings, western blotting was performed using various antibodies which demonstrated that SRC3, 53BP1 and the SRC3-interacting protein Creb binding protein (CBP) were specifically retained by the αSRC3 affinity column (Figure 1D).

**Figure 2:**
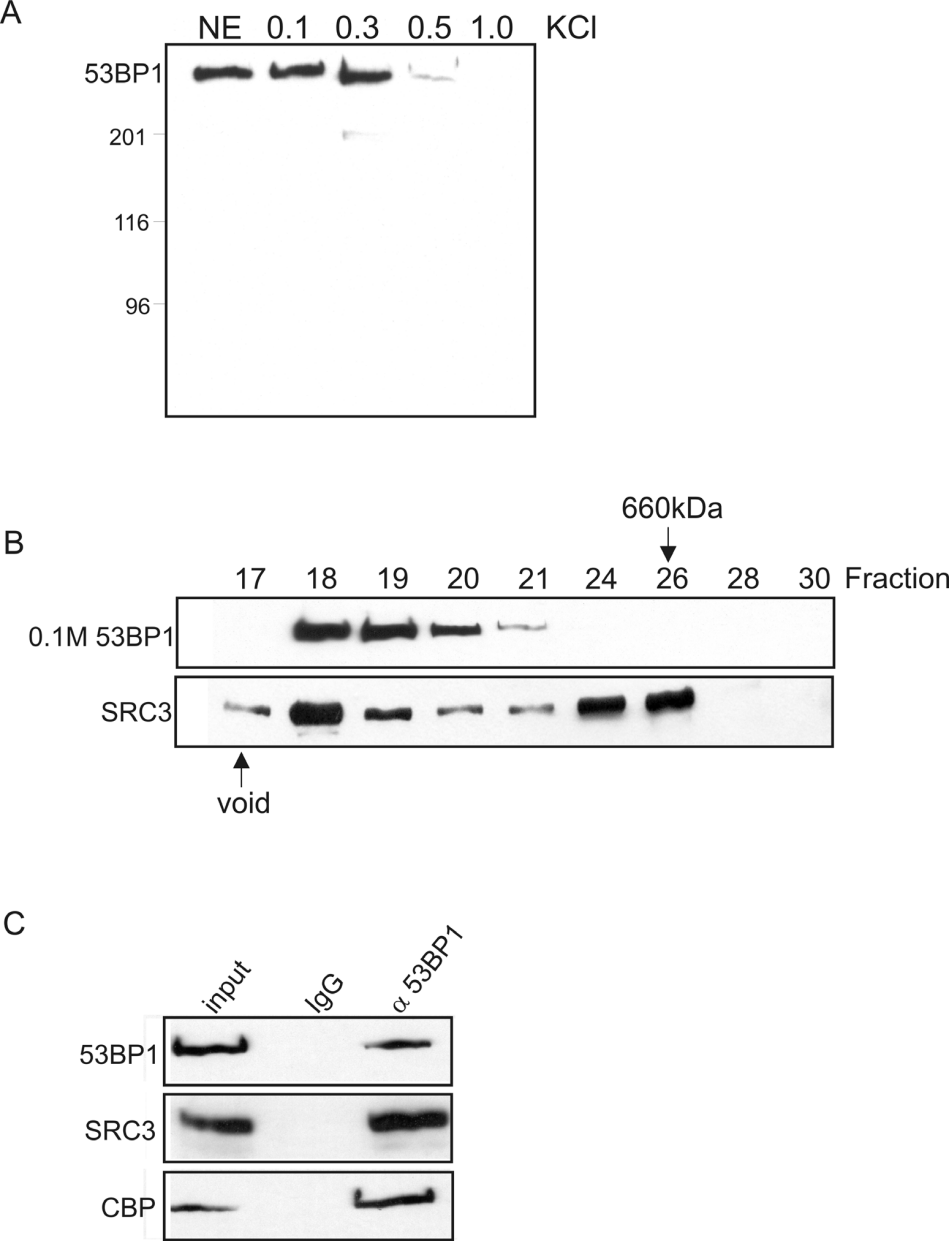
**Purification of 53BP1 from HeLa nuclear extracts**. (A) Phosphocellulose chromatography of HeLa cell nuclear extracts. Nuclear extracts were fractionated by p11 phosphocellulose chromatography. Bound proteins were eluted by increasing concentrations of KCl and analyzed for 53BP1 by western blotting. (B) Gel filtration chromatography of 53BP1 and SRC3. The 0.1 M phosphocellulosose fraction was concentrated and purified by gel filtration chromatography. Fractions were collected and assayed by western blotting using α53BP1 and αSRC3 antibodies. (D) Affinity purification of 53BP1. The 0.1 M 53BP1 gel filtration fractions containing SRC3 and 53BP1 were pooled and passed through an α53BP1 affinity column. Bound proteins were eluted with 100 mM glycine (pH 3.0), fractions were collected and aliquots were analyzed using the specific antibodies indicated on the left.

**Figure 3:**
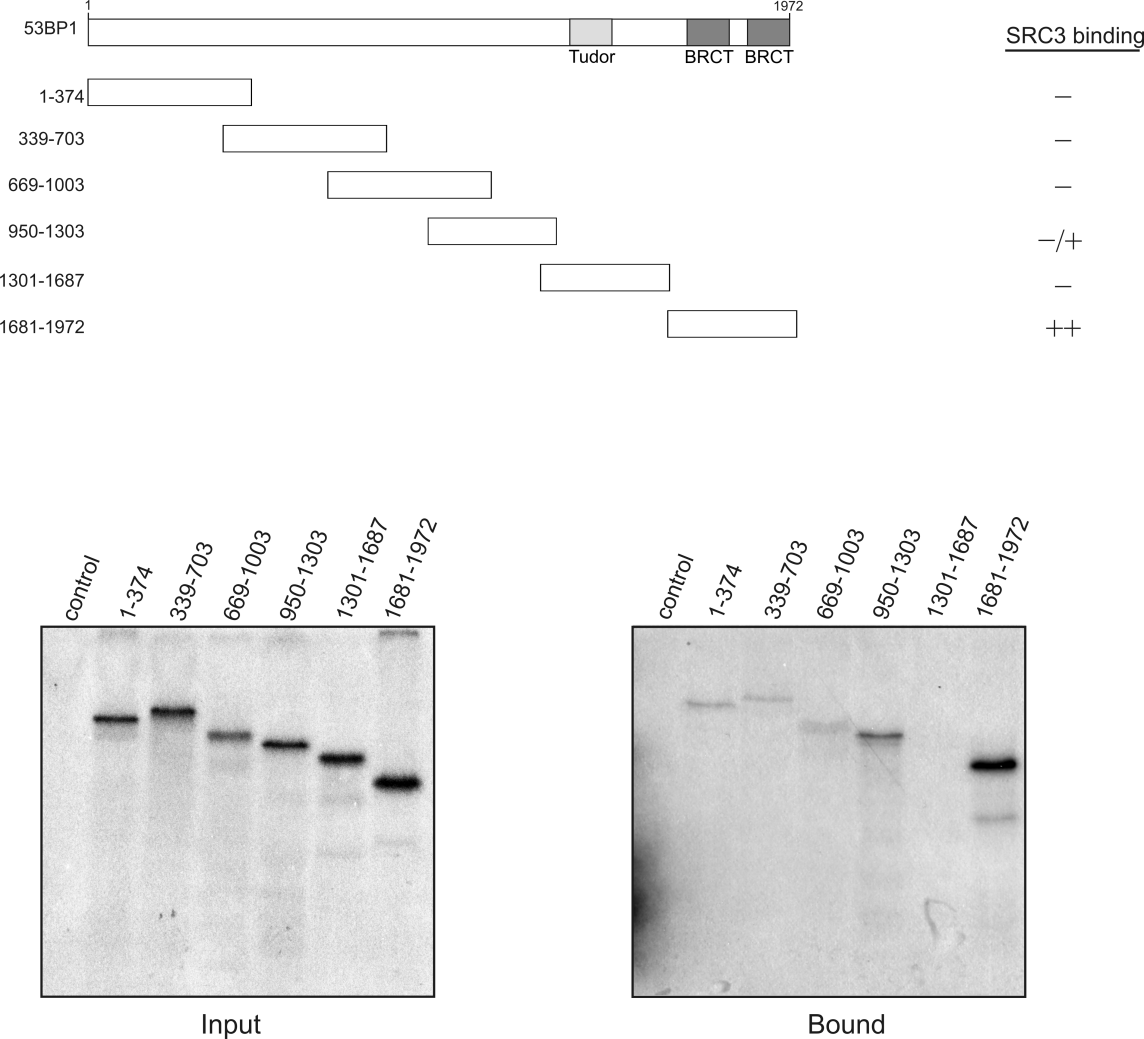
**SRC3 interacts with the carboxy terminus of 53BP1 *in vitro***. Various regions of 53BP1 were *in vitro *translated in the presence of^35^S-methionine and incubated with recombinant full length SRC3. The resulting complexes were isolated using FLAG Sepharose and analyzed by SDS-PAGE and fluorography. The input gel displays approximately 30% of the total amount of protein used for the interaction assay.

To further confirm the association between SRC3 and 53BP1, the purification protocol shown in Figure 1A was repeated using α53BP1 affinity column in place of the αSRC3 affinity column. Fractionation of HeLa cell nuclear extracts by phosphocellulose chromatography demonstrated that 53BP1 was found in both the 0.1 M and 0.3 M salt fractions suggesting that 53BP1 may be found as a constituent of other protein complexes (Figure 2A). The 0.1 M KCl fraction, containing both SRC3 and 53BP1, was then subjected to further purification using gel filtration chromatography. Elution from the gel filtration column indicated that 53BP1 migrated with an estimated molecular mass of approximately to 1 to 2 MDa and partially overlapped with the elution profile of SRC3(Figure 2B). The fractions containing both 53BP1 and SRC3 were pooled and purified by affinity chromatography using the α53BP1 antibody crosslinked to protein A Sepharose. Western blotting of the affinity purified fractions using specific antibodies demonstrated that this 53BP1 fraction also contained SRC3 and CBP confirming that they are most likely found in the same complex (Figure 2C). To determine if 53BP1 was able to interact directly with SRC3, deletion mutants of various region of 53BP1 were generated by *in vitro *transcription and translation with [^35^S]-methionine and tested for interaction with purified recombinant SRC3 (Figure 3). These experiments indicated that the carboxy terminus of 53BP1, containing the BRCT domains, interacted with full length SRC3, and a weaker interaction was detected with the region corresponding to (aa 950-1303). These results suggest that the carboxy terminus of 53BP1 makes direct contact with full length SRC3.

**Figure 4:**
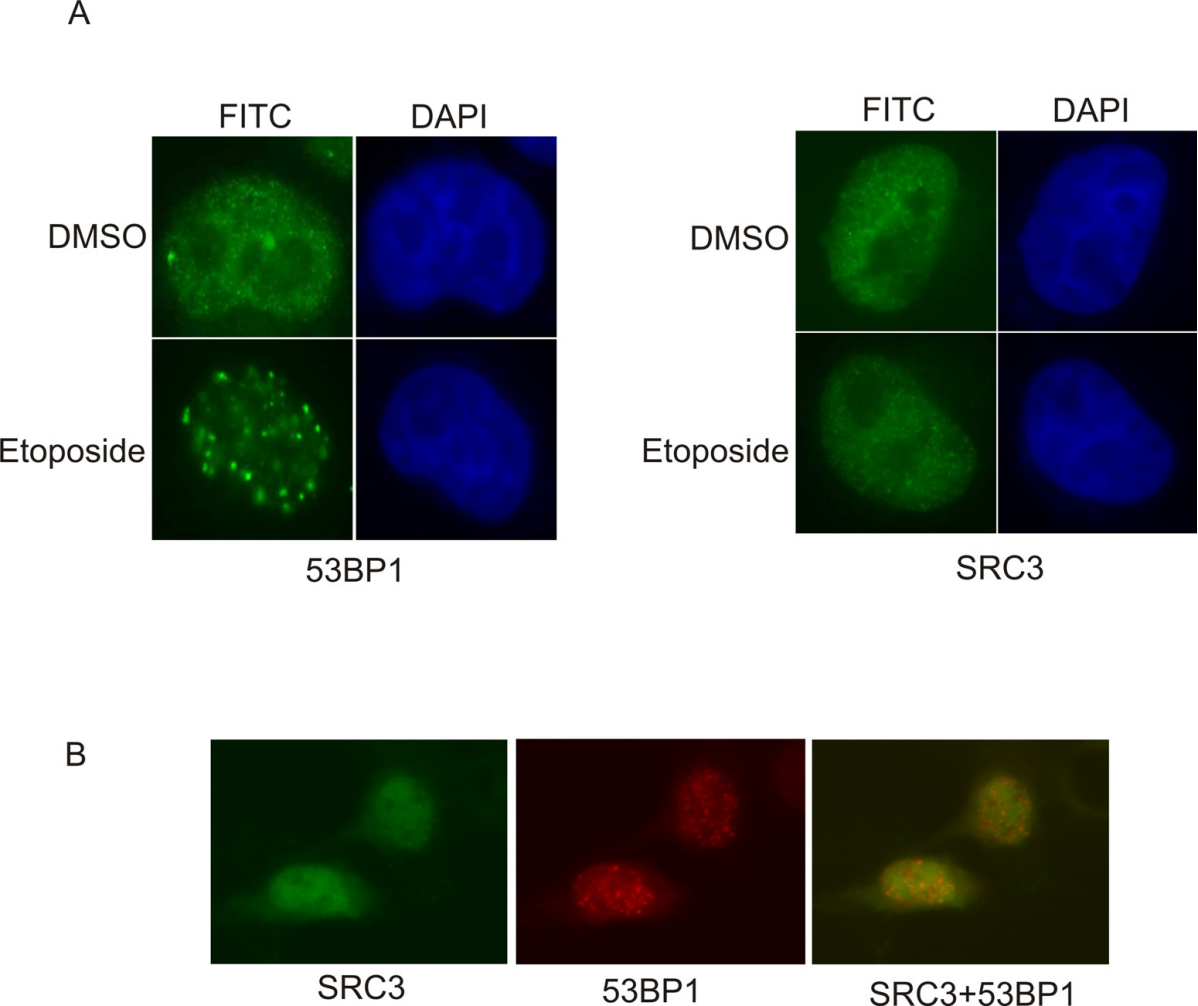
**Localization of 53BP1 and SRC3 in response to DNA damage**. (A) HeLa cells were treated with etoposide for 30 min followed by fixation and immunoflourescence analysis using either α53BP1 or αSRC3 primary antibodies. (B) HeLa cells were treated with 20 Gy γ-irradiation and allowed to recover for 30 min prior to staining with α53BP1 polyclonal antibody directly labelled with Alexafluor 594 or αSRC3 polyclonal antibody labelled with Alexaflour 488. DAPI, DAPI fluorescence marking the cell nuclei.

**Figure 5:**
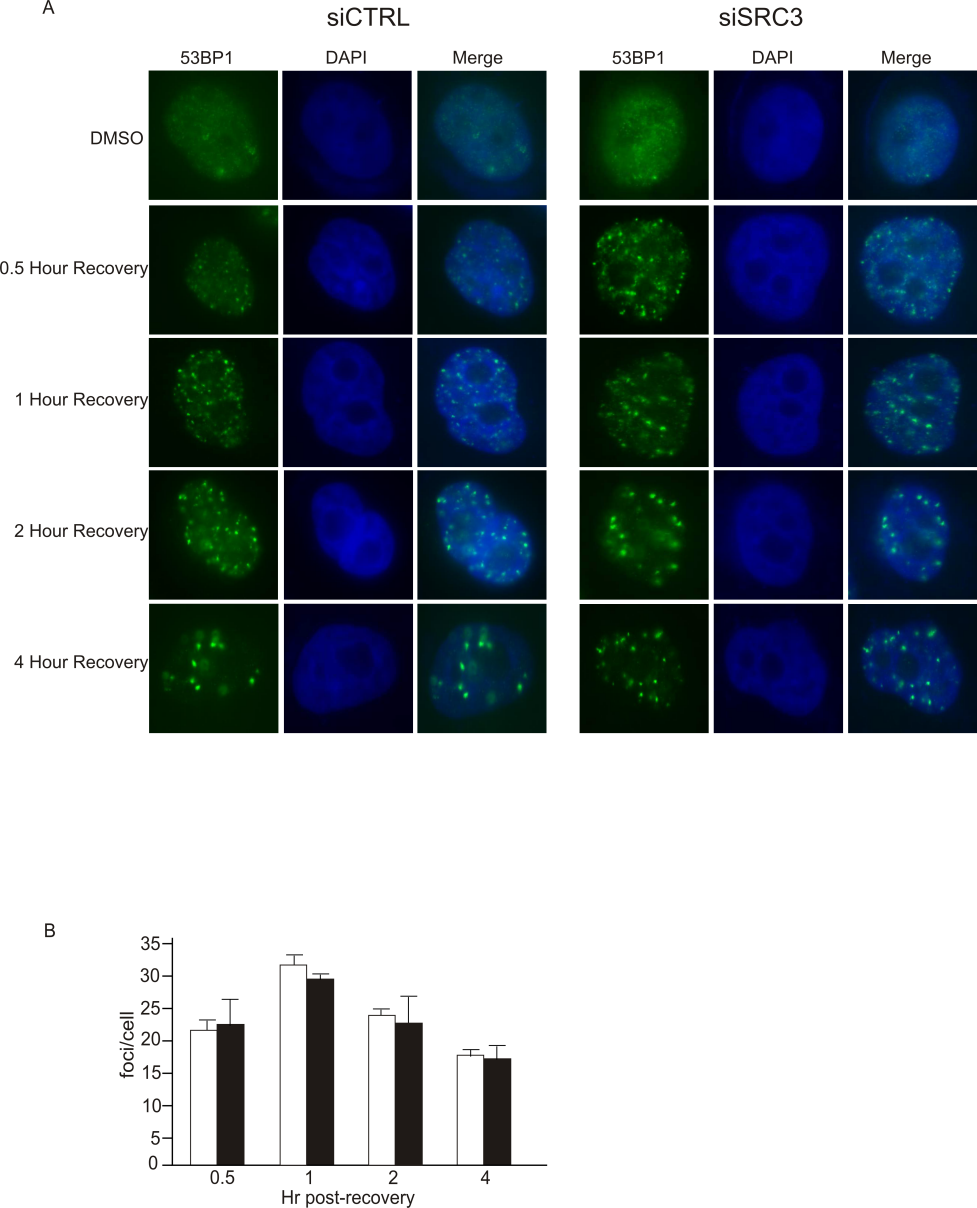
**Time course of 53BP1 relocalization following SRC3 knockdown**. (A) HeLa cells were transfected with control siRNA, or siRNA targeting SRC3. After 72 hr, the cells were stimulated with etoposide for 30 minutes, washed and then fixed and examined at various time points for 53BP1-dependent foci formation. (B)Plot of the average number of foci/cell at a given time point following etopiside treatment.

Numerous studies have established that the primary function of 53BP1 is in DNA damage response. When cells are exposed to agents which induce DNA double stranded breaks such as etoposide, 53BP1 rapidly relocalizes to discrete foci within the nucleus [29]. To define the potential role of the SRC3 in the DNA-damage response pathway, we investigated the effects of DNA damage on the localization of SRC3. For these experiments, Hela cells were treated with etoposide for 30 minutes, and the cells were allowed to recover, fixed and stained for either SRC3 or 53BP1. As shown in Figure 4A, in asynchronously proliferating HeLa cells both SRC3 and 53BP1 appear to be homogenously distributed throughout the nucleus. Upon treatment with 10 nM etoposide for 30 minutes, 53BP1 becomes localized to discrete foci. In contrast, the distribution of SRC3 remained unchanged when comparing the etoposide treated cells to the control cells. Distribution was also examined following gamma(γ) irradiation which again demonstrated that 53BP1 becomes localized to multiple foci whereas the distribution of SRC3 in the nucleus was homogenous (Figure 4B). Interestingly, some colocalization was evident at the 53BP1-containing foci when the images are merged. However, we believe that this is most likely not the result of an SRC3 response to the DNA damage because we did not observe any redistribution of SRC following treatment with etoposide. Furthermore, immunoaffinity purification of SRC3 indicated that 53BP1 copurifies with SRC3 regardless of whether the DNA had been damaged (data not shown). We also examined a potential regulatory role of SRC3 in the 53BP1-dependent response to etoposide following SRC3 knockdown. SRC3 levels were downregulated using siRNA and cells were treated with etoposide for 30 minutes then allowed to recover for various periods of time and foci formation were again monitored. Foci formation was clearly evident 30 minutes after etoposide treatment and the number of foci decreased in a time-dependent fashion (Figure 5A). Importantly, SRC3 knockdown using siRNA had no significant effect on the rate of recovery following etoposide treatment (Figure 5B). These results suggest that SRC3 is most likely not involved in the initial response to DNA damage.

**Figure 6:**
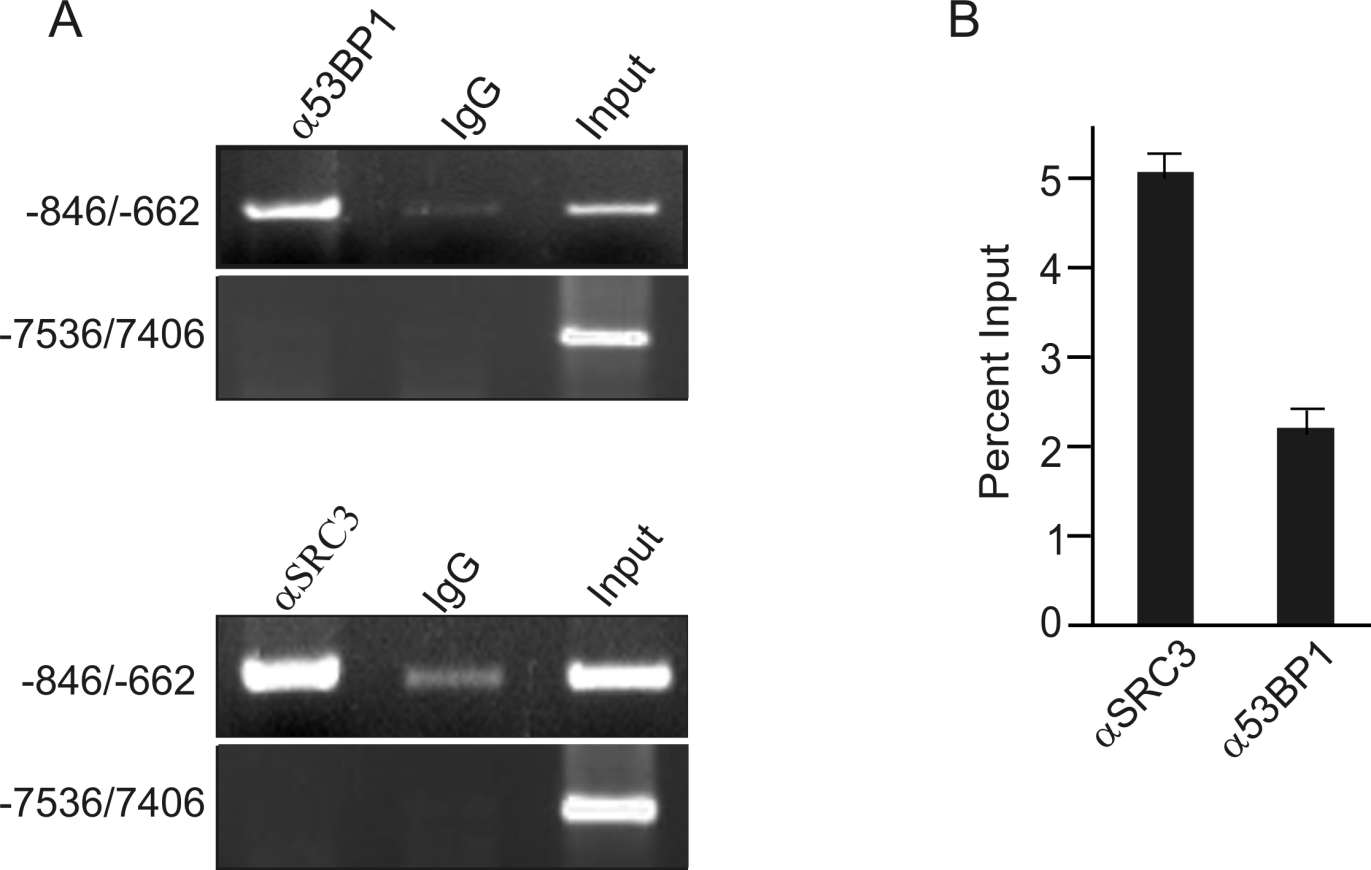
**ChIP analysis of of the BRCA1 promoter**. HeLa cells were crosslinked with 1% formaldehyde. Chromatin was then isolated and sonicated into approximately 1 kb fragments followed by immunoprecipitation with either control antibody (IgG), α53BP1 or αSRC3 antibody (IP) as indicated. The recovered DNA was then assayed by (A) conventional PCR or by (B) realtime PCR using pairs of oligonucleotides contained within the BRCA1 upstream region.

**Figure 7:**
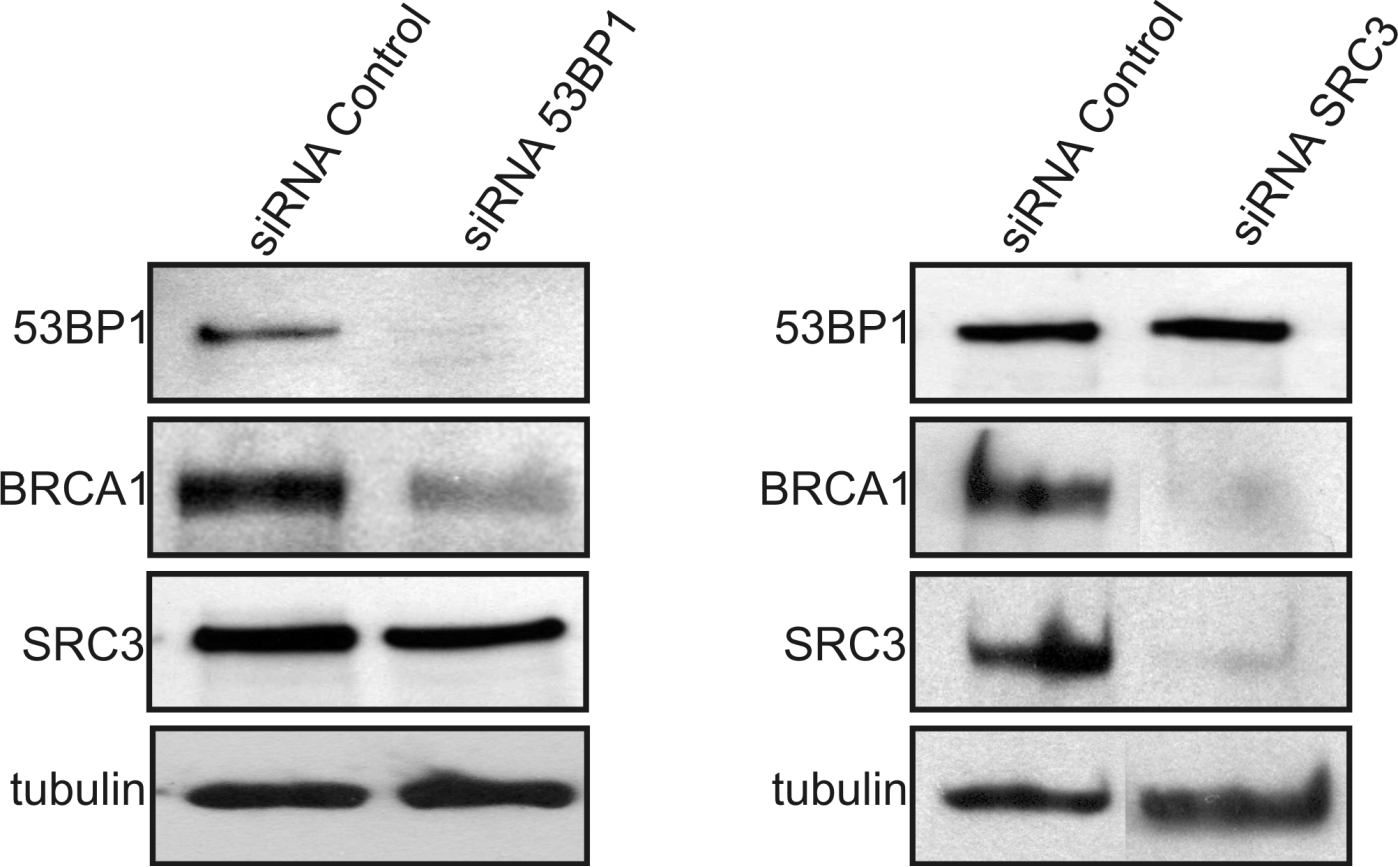
**SRC3 and 53BP1 are required for BRCA1 expression in HeLa cells**. HeLa cells were transfected with control siRNA, or siRNA targeting either 53BP1 or SRC3. After 72 hr. the cells were harvested and western blotting was performed on whole cell extracts using specific antibodies indicated on the left.

The lack of effect of SRC3 in the DNA damage response following etoposide treatment prompted us to examine whether the association between SRC3 and 53BP1 may be important for gene regulation. Recent studies have shown that a 53BP1-containing complex binds to an imperfect palindromic sequence found in the BRCA1 minimal promoter, and depletion of 53BP1 using siRNA suppresses both the activity of a reporter containing the minimal promoter and BRCA1 expression [30,31]. To determine if 53BP1 and SRC3 bind to a similar region of the BRCA1 promoter, we performed ChIP analysis in HeLa cells (Figure 6A and 6B). Both SRC3 and 53BP1 are enriched within the proximal promoter that contains the 53BP1 binding site described previously [30,31]. Finally, to assess if SRC3 or 53BP1 regulate BRCA1 protein expression, we used siRNA to knockdown SRC3 or 53BP1 in Hela cells. As shown in Figure 7, knockdown of 53BP1 or SRC3 resulted in significant decreases in BRCA1 protein levels suggesting that both proteins are required for expression of the BRCA1 gene. Collectively, these results suggest that the association between SRC3/53BP1 may represent a functional complex involved in transcriptional regulation of specific genes involved in DNA repair.

## Discussion

In the present study we have identified the tumour suppressor 53BP1 as a novel constituent of an SRC3 complex that also includes the protein acetyltransferase CBP. Silver staining of the affinity purified SRC3 complex detected at least 10 additional proteins; although the reproducibility of many of the purified proteins was not always consistent, they did not coelute from control antibody affinity columns. Therefore, we believe that they may play a complimentary role to SRC3 in response to specific stimuli. SRC3 belongs to the SRC family of proteins which function primarily as adaptor proteins involved in the recruitment and stabilization of protein complexes to upstream regulatory regions. Immunoaffinity chromatography has previously been used to identify a number of SRC-interacting proteins that include protein kinases, chromatin modifying proteins such as CBP/p300 as well as additional adaptor proteins [32-34]. While 53BP1 has not been previously identified as a SRC3-associated protein, many of the previous studies have used immunoaffinity purification using antibodies raised against different epitopes of SRC3 [32,33]. Consequently, it is conceivable that a specific protein-antibody interaction could result in masking specific interaction domains, or disrupt specific interactions, resulting in the purification of a different repertoire of interacting proteins.

Targeting protein acetyltransferases to specific sites in the genome has been shown to play an important role in double strand breaks (DSB) repair. The TRAP and TIP60 histone acetyltransferases are recruited to DSB and are required for H4 acetylation as well as the recruitment of additional DSB mediators including 53BP1 [35]. More recently, both CBP and p300 have been shown to play an important role in acetylation of histone H3 and H4 at DSB sites during non homologous end joining (NHEJ) which in turn facilitates recruitment of the SWI/SNF complex and consequently causing the chromatin to assume a more relaxed conformation [36]. Interestingly, both CBP and p300 have been found to associate with proteins involved in DNA repair and DNA damage response pathways, including 53BP1, suggesting that chromatin modification is a necessary prerequisite for the maintenance of genome integrity [33]. Finally, recent studies have implicated 53BP1 in NHEJ and V9(D)J recombination through a mechanism involving changes in chromatin mobility [37].

Based on our fractionation studies using 53BP1 antibody, 53BP1 consistently cofractionated into 2 distinct protein complexes following P11 chromatography. One complex eluted at a lower salt concentration contained both SRC3 and CBP, and a second complex which eluted at a higher salt concentration and does not contain SRC3 (data not shown). 53BP1 was first identified in a screen for p53-interacting proteins and was initially characterized as a coactivator of p53-dependent transcription [38]. 53BP1 contains tandem C-terminal BRCT motifs. Although the exact function of 53BP1 remains somewhat ambiguous it is believed to act as a scaffold or adaptor protein that coordinates DNA repair by facilitating recruitment of additional proteins required in the DNA repair process [39]. Mice deficient in 53BP1 exhibit a number of pleiotropic abnormalities including growth and immune deficiencies, extreme sensitivity to radiation and are cancer prone [40]. Importantly, cells from 53BP1-deficient mice exhibit intrinsic defects in genomic stability indicating that 53BP1 is a tumour suppressor protein [40]. When cells are exposed to genotoxic insults that cause DNA double stranded breaks, 53BP1 becomes localized to discrete nuclear foci which appear with varying degrees of rapidity and colocalize, in part, with other known components of the DNA damage response pathway [41,42]. The region of 53BP1 required for focal recruitment has been mapped to amino acids 1220-1711 and encompasses tandem tudor domains. The tudor domain has been shown to interact with methylated lysines and arginines and suggests that recruitment of 53BP1 to chromatin during DSB may proceed through multiple histone modifications [37]. For example the tudor domain of 53BP1 has been shown to interact with dimethylated H3K79 and H4K20 and studies have demonstrated a prerequisite for H3K79 dimethylation in the DNA damage response [43,44]. Interestingly, SRC3 is methylated by CARM1 *in vivo *and *in vitro*[14]. However, the region of 53BP1 that interacts with SRC3 contains the BRCT domains, not the tudor domains so it is unlikely that the association between 53BP1 and SRC3 is methylation dependent.

We did not find any evidence for a direct role for SRC3 in the DNA damage response, based on immunoflourescence analysis. While 53BP1 clearly relocalized to discrete foci in response to etoposide treatment, the distribution of SRC3 remained ubiquitous although some colocalization was observed within the foci following treatment with γ irradiation. Furthermore, knockdown of SRC3 using siRNA had no significant effect on the recovery rate following short term treatment with etoposide. While we cannot fully exclude a role for SRC3, based on this assay we believe that SRC3 is unlikely to play a direct role in the initial response to DNA damage. However, we have not examined other DSB mechanisms such as NHEJ [45]. In addition, there may be a level of redundancy with other SRC proteins which may compensate for any effect SRC3 may have in DNA damage response.

Our data suggests that 53BP1 plays a role in gene regulation and that the association between SRC3 and 53BP1 may be important for modulating the transcriptional response of the BRCA1 gene. A recent study has provided supporting evidence for this mechanism as 53BP1 may directly regulate gene transcription by targeting the BRCA1 promoter [30]. In vitro gel shift analysis has demonstrated that a 53BP1-containing complex binds to a consensus binding site found within the proximal promoter region of BRCA1 and ChIP analysis has shown that 53BP1 is targeted to the BRCA1 promoter [30]. Furthermore, depletion of endogenous 53BP1 in U2OS cells is associated with decreased BRCA1 mRNA and protein expression [30,37]. We have shown in Hela cells that both 53BP1 and SRC3 are found within the same approximate region of the BRCA1 promoter based on ChIP analysis. Importantly, downregulation of SRC3 or 53BP1 resulted in a similar loss of BRCA1 expression in HeLa cells.

The BRCA1 gene is a tumour suppressor that plays an essential role in multiple functions including DNA repair and transcriptional regulation and is regulated by a complex network of DNA binding proteins and coactivators [46,47]. Germline mutations in BRCA1 are relatively rare in sporadic forms of breast cancer although deregulation in BRCA1 expression is common [48]. In the majority of these cases the BRCA1 promoter is not hypermethylated suggesting that altered transcriptional regulation of the BRCA1 gene may play a causal role in this type of cancer. Upregulation of BRCA1 expression has also been shown to occur in response to 17β-estradiol in various breast cancer cell lines and in overectimized mice [49,50], and BRCA1 levels increase during puberty and pregnancy when estradiol levels peak [51]. Furthermore, treatment of MCF7 breast cancer cells with 17β-estradiol results in recruitment of ERα and p300 to the BRCA1 promoter that is, in part, mediated by AP1 [49,50]. It is well established that SRC3 interacts with both the ligand-bound ER and p300 with high affinity [1]. Thus, while we have not mapped the BRCA1 binding sites in detail, it is conceivable that in some cell types SRC3 and 53BP1 are recruited to the BRCA1 promoter as part of a larger complex involving the ER and as well as other transcription factors.

Although the biological significance of our findings remains to be elucidated, BRCA1 expression is regulated in a cell cycle dependent manner with the induction of BRCA1 peaking before the onset of DNA replication [52-54]. Consequently increases in BRCA1 expression resulting from SRC3 overexpression in some breast cancers may lead to aberrant activation of S-phase checkpoint proteins such as p53.

## Conclusion

SRC3 and 53BP1 are part of a complex which targets the BRCA1 gene promoter. Furthermore, both SRC3 and 53BP1 are required for BRCA1 expression suggesting that the SRC3/53BP1 complex plays a role in DNA damage by regulating the activity of a subset of target genes involved in DNA repair.

## Methods

### Antibodies and Reagents

The antibody to SRC3 was generated from a His-tagged recombinant protein and has been described previously [1]. The antibody to 53BP1 was generated from a His-tagged recombinant protein which was generated by digesting a cDNA plasmid containing the human 53BP1 protein with Bam HI and MScI and the corresponding fragment was subcloned into PQE31 vector (Qiagen). The plasmid was then transformed into M15 bacterial cells grown to an OD of approximately 0.7 and protein production by adding 0.5 mM IPTG. The recombinant protein was then purified using Ni-Agarose according to the manufacturers instructions (Qiagen) and was subsequently injected into rabbits for antibody production. The SRC3 and 53BP1 antibodies were purified by affinity chromatography using Protein A Sepharose according to standard procedures. All other antibodies were from Santa Cruz Biotechnology. The siRNA used in this study was obtained from Dharmacon. The siRNA against 53BP1 was GCCAGGUUCUAGAGGAUGAdTdT. Knockdown of SRC3 was performed using the SMART pool siRNA J-003759. The primers used for PCR analysis of the BRCA1 promoter were as follows, forward: GCCATTGATTGGTGGAGATT (-846), reverse: CGAGTCTCGGGCAAGTAGTC (-662), and the BRCA1 upstream primers were as follows, forward: TGCAACACACCCAGAGTACC) (-7536) and reverse: TTGCATTGTTCTGACCACCA (-7406).

### Purification of SRC3 and 53BP1

For small scale scale purifications, HeLa cells were typically grown on 150 mm dishes to 80% confluency prior to harvesting. For larger scale purifications, 20 litres of cells were grown to mid-log phase prior to harvesting and nuclear extracts were prepared according to standard methods [55]. The nuclear extract was dialyzed against buffer A [20 mM Tris (pH 7.9), 100 mM KCl, 0.5 mM EDTA, 0.5 mM EGTA, 10% glycerol, 0.5 mM DTT, 0.2 mM PMSF and 5 μg/ml each of leupeptin, aprotinin and pepstatin] and this fraction was loaded onto a P11 phosphocellulose column preequilibrated in the same buffer. The flowthrough was collected and the column was washed sequentially with increasing KCl concentrations. The relevant fractions were pooled, and the proteins were precipitated with 20 to 60% ammonium sulphate. The precipitated proteins were resuspended in a small volume of buffer A and then dialyzed against the same buffer to remove residual ammonium sulphate. This was then passed through a Sephacryl S300 column and fractions corresponding to either the SRC3 or 53BP1 containing peak were pooled, concentrated and dialyzed against buffer A containing 100 mM KCl without DTT.

For affinity purifications, the SRC3 and 53BP1 antibody were crosslinked to Protein A sepharose using dimethylpalmilidate (DMP) according to standard procedures [56]. Fractions from the gel filtration step were loaded onto the affinity column at a flow rate of 0.2-0.5 ml/min and the flowthrough was collected and reloaded on the the column five times prior to elution of the bound proteins with 100 mM glycine (pH 2.8). For mock purification experiments, samples from the gel filtration step were loaded onto protein A sepharose alone, or protein A sepharose crosslinked to an irrelevent antibody.

Subcellular fractions of cells were prepared according to standard methods [55].Western blotting was performed as described previously [1]. Normally 20 μg of protein was loaded and analyzed by standard SDS-PAGE, transferred to nitrocellulose and detected by ECL according to the manufacturers recommendations (Amersham).

### Mass Spectrometry

Purified complexes were separated by 7% SDS-PAGE and then stained with colloidal blue for 1 h followed by destaining in 25% MeOH for an additional 2 h. The p300 band was excised and cut into 1 mm pieces. The gel pieces were washed twice in a 50% CH_3_CN solution for 5 min followed by two washes with a 250-ml solution consisting of 50% CH_3_CN, 50 mM NH_4_HCO_3 _for 30 min. The gel pieces were lyophilized, rehydrated in 10 mM NH_4_HCO_3_, pH 8.5 containing 0.1 mg/ml trypsin (Roche Molecular Biochemicals) and incubated overnight at 37°C. The tryptic fragments were extracted by two 30 minute washes with a solution containing 60% CH_3_CN and 10% trifluoroacetic acid. The combined solutions were lyophilized using a Speedvac, resuspended in 20 ml of 0.5% trifluoroacetic acid solution and the peptide suspensions were purified using a ZipTip (Millipore) cartridge. Samples were then analyzed by LC-MS at the Centre d'innovation Génome Québec (McGill University)

### Expression and Purification of Recombinant Proteins

FLAG-tagged SRC3 was generated by subcloning SRC3 cDNA into the pFastbac vector (InVitrogen) and recombinant proteins were expressed using the Bac-to-Bac baculovirus expression system according to the manufacturers instructions. Epitope-tagged proteins were prepared by infection of SF9 cells with the appropriate recombinant baculovirus followed by immunoaffinity chromatography with anti-Flag M2 affinity resin essentially as described [14]. Proteins were eluted with 20 mM Tris buffer pH 7.9, 100 mM KCl, 10% glycerol, 0.5 mM EDTA and 0.2 mg/ml of the appropriate peptide competitor. Proteins were then frozen and stored at -80°C.

### In vitro interaction assays

Various regions of 53BP1 cDNA were PCR amplified and subcloned into the Pcite vector (Novagen). Approximately 2.5 μg of plasmid was used for each *in vitro *transcription/translation reaction in the presence of 2 μl^35^S-Met according to the manufacturers instructions (Promega). The reaction was allowed to proceed for approximately 75 minutes. 1 μg of epitope-tagged full length SRC3 was incubated with each *in vitro *translated reaction (100,000 cpm) at 4°C for 2 hr in 250 μl of PI buffer consisting of 20 mM Tris (pH 7.9), 300 mM KCl, 0.5 mM EDTA, 1 mM MgCl_2_, 1 mM DTT, 10% glycerol, 0.1 mM PMSF and 1 mg/ml BSA. Each reaction was then incubated with 25 μl FLAG-Sepharose for 30 minutes at 4°C then washed 5× with PI buffer. After the final wash, the beads were resuspended in 30 μl 2× SDS sample buffer and analyzed by SDS-PAGE. The gel was then fixed, incubated with Amplify (Amersham) for 30 minutes dried and exposed to film at -80°C for approximately 96 hr.

### Immunostaining and microscopy

Hela cells were grown on cover slips in 6 well plates and treated with DMSO or etoposide for the indicated times and were then fixed for 15 min with 4% formaldehyde in PBS followed by a 10-min incubation with 0.1 M glycine in PBS. Cells were then permeabilized with 0.5% Triton X-100 in PBS for 10 minutes. Immunostaining was performed with SRC3 antibody (1:400 dilution) or 53BP1 antibody (1:400 dilution) and fluorophore-conjugated Donkey secondary antibodies (CY3, FITC) (Jackson ImmunoResearch Laboratories). For the experiments examining the effects of γ-irradiation, affinity purified SRC3 and 53BP1 antibodies were directly labeled with Alexaflour 594 and 488, respectively according to the manufacturers instructions (Invitrogen). Immunflourescence was then performed with the conjugated primary antibodies. Epifluorescence imaging was performed on an Axiovert 200 M inverted microscope equipped with an Apotome (Carl Zeiss) using appropriate fluorophore-specific filter sets. Z-series images (63 × magnification) were acquired at 0.5-μm intervals and processed with Axiovision software and Adobe Photoshop. Fluorescence intensity plots were obtained by performing a line scan bisecting the cell using Axiovision software.

### Chromatin immunoprecipitation assay

HeLa cells were cross-linked with 1% formaldehyde at room temperature for 10 min. Cells were washed twice with ice-cold PBS containing 0.5 mM EDTA and harvested. Cells pellets were lysed in 0.3 ml of cell lysis buffer (50 mM Tris-HCl [pH 8.1], 10 mM EDTA, 1% SDS, and protease inhibitors) and incubated on ice for 10 min. Cell lysates were sonicated to yield DNA fragments ranging in size from 750- to 1,000 base pairs. Approximately 450 μg of the cross-linked, sheared chromatin solution was used for immunoprecipitation with. A small portion of each sample was saved as input DNA (5%). Supernatants were diluted 10-fold in dilution buffer (20 mM Tris-HCl [pH 8.1], 1% Triton X-100, 2 mM EDTA, 150 mM NaCl, and protease inhibitors) and precleared with 60 μl of 50% slurry protein A-Sepharose containing 2.5 μg of sheared salmon sperm DNA for 2 h at 4°C. Immunoprecipitation was performed overnight at 4°C with 1.5-4 μg of the antibodies. 60 μl of protein A-Sepharose containing 2.5 μg of salmon sperm DNA per ml was added to the solution and incubated for 1 h at 4°C. The beads were washed one time with wash buffer I (0.1% SDS, 1% Triton X-100, 2 mM EDTA, 20 mM Tris-HCl, 150 mM NaCl), wash buffer II (0.1% SDS, 1% Triton X-100, 2 mM EDTA, 20 mM Tris-HCl, 500 mM NaCl), wash buffer III (0.25 M LiCl, 1% NP-40, 1% Na-Deoxycholate, 1 mM EDTA and 10 mM TrisHCl). Immunocomplexes were extracted twice with 200 μl elution buffer (1% SDS and 0.1 M NaHCO_3_). NaCl was added to a final concentration of 200 mM and the cross-linking was reversed by heating at 65°C overnight. The DNA was purified using Qiagen PCR purification spin columns. For analysis by conventional PCR, conditions were as follows: initial denaturing cycle of at 94°C for 3 min, followed by 35 cycles of 94°C for 30 sec, 52°C for 30 sec and 72°C for 1 min, and a final elongation step of 72°C for 10 min.

For some experiments, DNA isolated from ChIP experiments was subjected to quantitation by real time PCR using Brilliant SYBR green master mix (Stratagene; 600548). Primers were identified using the Primer Express program (Stratagene) and tested to establish optimum reaction conditions. Reactions were performed in a 25 μl volume according to manufacturer's recommendations. The reaction was carried out and measured using Mx3000P realtime instrument. The nonimmune IgG copy number was subtracted from IP DNA copy number. The resulting IP copy number was normalized against the total input DNA by dividing the IP by input and expressing the IP as a percentage of the input DNA. All measurements were done in duplicates and an average Ct value was used to calculate copy number. Two independent realtime reactions were done for each experiment.

## Authors' contributions

DC and RM performed the immunoflourescence experiments and the *in vitro *interaction experiments. GT performed the ChIP assays. NC performed confirmatory analysis of the knockdown experiments, as well as repeating the knockdown experiments with the appropriate controls. MI assisted with the protein purifications, performed western blots and confirmed the siRNA knockdown experiments. MT was involved in all theoretical work and provided input regarding experimental design. Was involved in all theoretical work, wrote the manuscript and performed the purifications. All the authors have read and approved the final manuscript.
